# Development and Characterization of Mechanically Durable Silicone-Polythiourethane Composites Modified with Tetrapodal Shaped ZnO Particles for the Potential Application as Fouling-Release Coating in the Marine Sector

**DOI:** 10.3390/ma11122413

**Published:** 2018-11-29

**Authors:** Haoyi Qiu, Iris Hölken, Anna Gapeeva, Volkan Filiz, Rainer Adelung, Martina Baum

**Affiliations:** 1 Functional Nanomaterials, Institute for Materials Science, Faculty of Engineering, Kiel University, Kaiserstr. 2, D-24143 Kiel, Germany; haq@tf.uni-kiel.de (H.Q.); ih@phi-stone.de (I.H.); ang@tf.uni-kiel.de (A.G.); ra@tf.uni-kiel.de (R.A.); 2 Phi-Stone AG, Kaiserstr. 2, D-24143 Kiel, Germany; 3 Institute of Polymer Research, Helmholtz-Zentrum Geesthacht, Max-Planck-Str. 1, 21502 Geesthacht, Germany; volkan.filiz@hzg.de

**Keywords:** polymer blend, fouling-release coating, tetrapodal ZnO, PDMS

## Abstract

Ecological considerations strongly necessitate the development of environmentally friendly antifouling paints. A promising alternative to biocide containing antifouling paints are fouling-release coatings, which are non-toxic and designed to prevent permanent attachment of marine organisms to the surface, due to their low surface energy. However, these coatings suffer from insufficient mechanical properties, which make them unsuitable for mechanically stressed surfaces e.g., on ship hulls. To overcome those obstacles, polydimethylsiloxane (PDMS)-polythiourethane (PTU) composites modified with tetrapodal shaped micro-nano ZnO particles (t-ZnO) were produced and characterized by evaluating the surface energy, mechanical properties, and fouling-release performance. Among all variations, PTU/1 wt.% PDMS composites with 1 wt.% t-ZnO particles possess superior properties for applications as fouling-release coatings for maritime purposes.

## 1. Introduction

The undesired growth of marine organisms on surfaces subjected to water is generally known as biofouling, and as it brings along several ecological and economical drawbacks, it represents one of the major challenges in the marine industry [1,2,3]. This implies overall increased weight on the ship and elevated hydrodynamic drag resistance, which results in additional fuel consumption of up to 40% and in turn leads to increased greenhouse gas emissions and critical environmental pollution [4,5,6]. Another important aspect of biofouling involves the deterioration of materials, as a consequence of biocorrosion. The impact of this effect is widespread and ranges from stationary maritime buildings, like basements for offshore wind turbines, to ship hulls, to cooling systems of power plants, to fuel tanks and pipes [7,8,9,10,11].

Since ancient times, humans have tried to find efficient solutions for preventing biofouling on ships and on submerged maritime buildings [12]. In this context, the development of tributyltin (TBT)-based antifouling coatings in the 1950s led to a convincingly marketable and effective product. The principle of these coatings is based on biocidal additives, which are constantly released from a degrading organic polymer matrix, a so-called self-polishing coating [13]. However, a report by Alzieu [14] showed the negative impact of these biocides on the natural environment, based on a study on the embryogenesis of oysters conducted in France during the 1970s. As a result of even very low TBT concentrations, it was shown that the oysters suffered from complete lack of reproduction as well as cell calcification anomalies [14]. Hence, global regulations were initiated in 2003 by the international maritime organization (IMO), including the complete prohibition of TBT-containing coatings [15]. A common alternative solution to those harmful coatings was provided by copper-based self-polishing paints, combined with herbicidal additives. However, as they also create negative impacts on the marine environment, prohibition against them is also pending [16]. In order to develop an antifouling paint without a negative ecological impact, current approaches tend primarily towards hindering fouling organisms from attaching to substrates by tuning the surface properties in such a way that attachment is unfavorable [5]. This can be realized by developing either superhydrophilic [17] or superhydrophobic [18] surface properties, as these modified surfaces are known to act as self-cleaning materials like, e.g., anti-fog glasses [19] and most waterproofed clothing [20]. However, many proteins can easily adapt to both kinds of surface properties by using either their hydrophobic cores or their hydrophilic coronas to attach and cause biofouling on basically any type of surface [20]. Using fouling-release coatings to deal with this effect has raised attention within the last couple of years. Those are based on the idea of minimizing adhesion forces in between the substrate and the attached organisms, so that in principle they can be removed by hydrodynamic forces provided by a moving ship [4,21]. These coatings are usually composed of silicone elastomers or polytetrafluoroethylene (PTFE) components. The silicone elastomers are a unique material class, as they combine an inorganic –Si–O– backbone with organic radicals attached to the silicon atom. This structure provides an extremely low elastic modulus caused by the large Si–O–Si bond angles, as well as very low surface energies in the range of 21–22 mN/m [22]. However, silicone-elastomers suffer from weak mechanical robustness combined with poor adhesion characteristics to the substrate, which make them unsuitable for mechanically stressed surfaces like ship hulls [6,23]. To maintain the advantageous surface properties of silicones and simultaneously gain the desired mechanical stability and adhesion to the substrate, the development of phase-separated composites based on silicones and polyurethanes provided enormous progress [24,25,26,27,28,29]. As a mechanically durable polymeric matrix, thermoset polyurethanes (PU) offer great potential, due to their highly cross-linked structure, which results in high tensile strength as well as enhanced abrasion resistance [30]. Due to these properties, PU is a well-established material for ship hull coatings, but with a crucial lack of antifouling properties [31]. To ensure environmental compatibility, the polymeric matrix should also be abled to be processed without the utilization of harmful volatile organic compounds (VOC), which are widely used in the coating industry [32]. These requirements are fulfilled by the two-component aliphatic full-solid polythiourethane (PTU) thermoset, which forms a highly cross-linked polymer matrix by polyaddition of four reactive SH-groups. Additionally, this aliphatic polymer is UV-stable due to the lack of benzene rings, which are characteristic in aromatic PUs [33]. To modify the surface properties as well as to obtain optimum mechanical stability, ceramic t-ZnO particles were incorporated into the polymeric matrix, which has already been subject of earlier studies [34,35,36,37]. It has been suggested that the mechanical reinforcement of polymer composites in combination with those particles is partly due to their three-dimensional morphology [34]. Beyond this, PTU/t-ZnO composites have already proven to possess convincing low-fouling features and impressive resistance to biocorrosion in earlier experiments [36,37]. The aim of this work is to develop a fouling-release coating with superior mechanical properties, applicable to large-scale objects like ship hulls and submerged maritime structures. For this reason, a polymer blend, based on a highly durable PTU matrix polymer, reinforced by t-ZnO particles in combination with one-component silicone elastomer, was produced and investigated. The composites were characterized with respect to physico-chemical surface properties, mechanical tensile strength, and elongation at fracture. The fouling-release features of the composites were investigated by peel-off tests, long-term static immersion field-tests in the Baltic Sea, and subsequent barnacle removing experiments.

## 2. Materials and Experiments

### 2.1. Materials

The components of the matrix polymer, polythiourethane (PTU), aliphatic 1,6-diisocyanatohexane (HDI) with a density of 1.15 g/cm^3^, and tetrafunctional pentaerythritoltetrakis (3-mercaptopropionate) (PETMP) with a density of 1.28 g/cm^3^ were supplied by Fluid & Prozesstechnik (Waltershausen, Germany). The silicone component for the polymer blend, polydimethylsiloxane (PDMS) Elastosil E43 with a density of 1.09 g/cm^3^, was purchased from Wacker Chemie AG (Munich, Germany). The t-ZnO particles with a mean arm length of 50 µm were produced by the flame transport synthesis (FTS) approach [38] at the Faculty of Engineering, Kiel University, Kiel, Germany.

### 2.2. Sample Preparation

For all prepared formulations, the mixing ratio of 58 wt.% HDI to 42 wt.% PETMP was kept constant, whereas the PDMS amount was varied between 0 wt.%, 1 wt.%, 3 wt.%, 5 wt.%, 8 wt.%, and 10 wt.% and the t-ZnO amount varied between 0 wt.%, 1 wt.%, 3 wt.%, and 5 wt.%. To avoid curing of the formulations before complete preparation, PDMS and t-ZnO were pre-mixed with the appropriate amount of PETMP utilizing a Netzsch MiniMaster Disperser (Netzsch-Feinmachltechnik GmbH, Selb, Germany) for 15 min at the rotational speed of 6000 rpm. The HDI component was subsequently added and stirred-in by hand. The samples for surface characterization were prepared by casting the mixed polymer into silicone molds with dimensions of 80 mm × 80 mm × 5 mm. The samples for the peel-off tests were prepared with dimensions of 80 mm × 25 mm × 5 mm by casting, while PVC and AlMg3 substrates with the thickness of 3 mm were cut into the same dimensions as the references. The biofouling test samples were coated with the mixed polymer by paint roller on AlMg3 substrates with the dimensions of 80 mm × 80 mm, where the coating thickness was around 0.5 mm. All samples for surface characterization and biofouling tests were cured at room temperature for 5 days. For tensile tests, dog-bone-shaped tensile test specimens with a thickness of 1 mm, a width of 5 mm, and a length of 20 mm were used according to ISO 527, and all samples were cured at 85 °C for 24 h in an atmospheric furnace (Heraeus instruments, Hanau, Germany).

### 2.3. Instrumentations

For the evaluation of the mechanical performance of the polymer composites, tensile tests were performed using a Zwick 1445 universal tensile testing machine (Zwick GmbH und Co. KG, Ulm, Germany), with an initial load of 5 N and a constant strain rate of 5 mm/min. In order to investigate the influence of different parameters and additives on tensile strength and elongation at fracture, mean values with corresponding standard deviations were calculated from ten samples of each variation. For statistical evaluation, one-way analysis of variance (one-way ANOVA) followed by a Tukey test (significance level of *p* < 0.05) was performed by OriginLab (OriginLab Corporation, Northampton, MA, USA).

To characterize the surface properties of the samples, the surface free energy (SFE) was determined by measuring the contact angle with a Krüss DSA 100 system (Krüss GmbH, Hamburg, Germany) by the sessile drop method with a drop volume of 2 µL. The SFE and its polar and dispersive components were evaluated by measuring the contact angles of three liquids with varying surface tensions (SFE: water: 1000 mN/m, ethylene glycol: 47.5 mN/m, chlorobenzene: 33.6 mN/m). Three surfaces were investigated for each variation of the composite and ten measurements were performed on each surface. The contact angles were calculated by Young-Laplace fitting [39]. The SFE was calculated by the method of Owens, Wendt, Rabel, and Kaeble [40]. Mean values and corresponding standard deviations were calculated for each surface investigated.

To determine the chemical composition of the polymer blend in general and possible influences of the PDMS component on the polymerization reaction of the matrix polymer in detail, Micro-Raman-spectroscopy was performed on pure PTU, pure PDMS, and PTU/PDMS composite samples with a confocal Raman spectrometer (Witec Alpha 300RA, Ulm, Germany), incorporating an Ar laser of 532 nm wavelength. Three measurements on a 100 µm × 100 µm scanning area, 150 points per line, and 150 lines per image were conducted on each sample for error reduction.

The morphology and the surface roughness of the composites were imaged and measured by 3D Laser Scanning Confocal Microscope VK-X (Keyence Corporation, Osaka, Japan). The measurement light source was a red semiconductor laser with wavelength of 658 nm. Ten line-scans were conducted on each surface for the surface roughness measurement. For detailed elemental characterization, energy-dispersive X-ray (EDX) spectroscopy at an acceleration voltage of 18 keV was performed with a Zeiss Ultra Plus scanning electron microscope (SEM), equipped with an EDX-detector (Carl Zeiss AG, Oberkochen, Germany).

To evaluate the potential of the composite to be used as fouling-release coating, the 90° peel-off tests of commercially available adhesive tape (Tesa classic tapes, Tesa SE, Norderstedt, Germany), with width of 19 mm from different samples, were conducted with the Quick Test (Prüfpartner GmbH, Langenfeld, Germany), according to ISO 29862:2007. The adhesive tapes were pressed by covering a flat AlMg3 substrate with a uniform force of 150 N after being applied on the samples’ surfaces. The tests were performed at a loading speed of 360 mm/min. Measurement values with the initial and last length of 15 mm were discarded and the average adhesive force with an effective length of 50 mm was calculated. Six measurements per surface were performed, from which the mean values and corresponding standard deviations were calculated.

In order to investigate the fouling-release properties of the composites under realistic conditions, three specimens of each formulation were immersed into the Baltic Sea (Laboe harbor, Germany) with mean annual water temperature of 10.5 °C (max 20.6 °C, min −0.3 °C) and salinity of around 15 psu, in a depth of 0.5 m (Federal Maritime and Hydrographic Agency, Hamburg, Germany). Pure PTU, pure PDMS, PVC, and AlMg3 (a well-established material for ship building) were used as references. All samples were mounted on a PVC substrate by random arrangement and vertically submerged 0.5 m below the water surface. The experiment started in January 2017 and ended in December 2017. After one year of immersion, the barnacles on the samples were removed manually to evaluating the fouling-release properties of each surface in terms of barnacle adhesion. The surface appearance before and after barnacle removal was recorded by camera (Olympus TG-4, Olympus Corporation, Tokyo, Japan) for each sample.

## 3. Results and Discussion

### 3.1. Characterization of the Influence of the PDMS Amount on Mechanical Properties of the PTU/PDMS Polymer Blend

**Figure 1:**
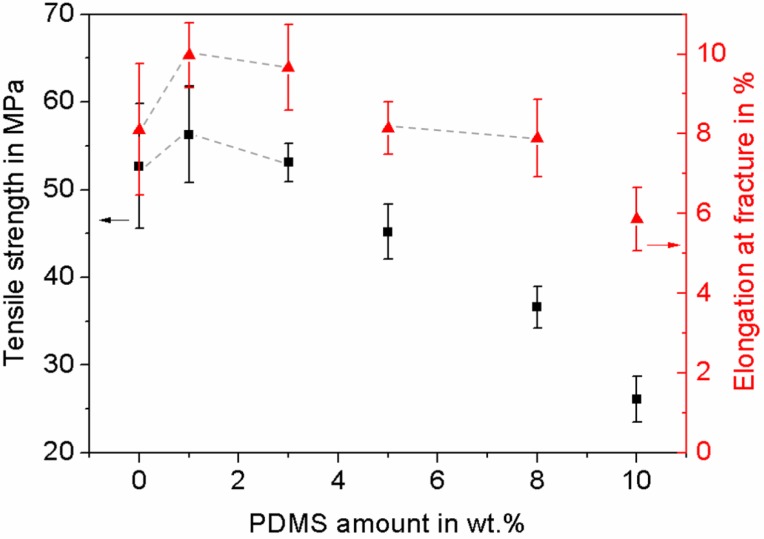
Tensile strength and elongation at fracture as functions of polydimethylsiloxane (PDMS) amount. Mean values and corresponding standard deviation of tensile strength and elongation at fracture are shown as a function of the PDMS amount. For statistical analysis of the mean values of tensile strength and elongation at fracture, a one-way ANOVA followed by a Tukey test was performed. Data points connected by gray dashed lines showed no significant differences in between each other (*p* < 0.05).

To investigate the influence of the PDMS amount on the overall mechanical performance of the composite, tensile tests were performed on samples with varying PDMS content (0 wt.%, 1 wt.%, 3 wt.%, 5 wt.%, 8 wt.%, 10 wt.%) (Figure 1).

The tensile strength as well as the elongation at fracture decreased corresponding to increasing PDMS content, except for the addition of 1 wt.% PDMS, which caused a slight increase in tensile strength of around 7%, and a stronger increase in elongation at fracture at more than 20%. However, statistical data analysis (one-way ANOVA, Tukey test, *p* < 0.05) showed no significant difference for the filling factors of 0 wt.%, 1 wt.%, and 3 wt.% t-ZnO. Modifying the composite with 8 wt.% and 10 wt.% PDMS content led to strongly reduced mechanical properties. Further investigations were focused on 1 wt.% PDMS, because this composition has optimal mechanical properties and lowest PDMS amount. In one of our previous studies, a higher tensile strength value of pure PTU was obtained [37], which can be attributed to a different sample preparation procedure, where the polymer was degassed. In this study the degassing step was omitted for PTU/PDMS blends in order to avoid agglomerations of PDMS. To make a proper comparison of the material properties between the investigated material variations, pure PTU was not degassed either, which resulted in the lower tensile strength value.

To give an explanation on the increase in elongation at fracture and the maintained tensile strength at low PDMS amounts, it can be assumed that the elastic deformation of the composite material is dominated by the mechanical properties of the PTU matrix, while the plastic deformation is already strongly influenced by the high elasticity of the PDMS component [41]. At higher PDMS amounts, the overall mechanical performance of the composites deteriorated. The silicone can be regarded as the origin of defects, strongly influencing the tensile response and therefore also material failure [35,36,37].

### 3.2. Physico-Chemical Characterization of the PTU/PDMS Polymer Blend

Besides the mechanical features, the application of the PTU/PDMS composites as low-fouling coating in the marine sector exhibits surface properties comparable to those of the well-known fouling-release material group of silicones. For this reason, contact angle measurements were conducted and the surface free energy (SFE) with its polar and dispersive fraction was calculated.

For the PTU/PDMS composite, the SFE as well as the dispersive and polar fractions resemble those of pure PDMS. It could therefore be shown that the SFE and its polar and dispersive fractions of pure PDMS were transferred to the composite material.

**Figure 2:**
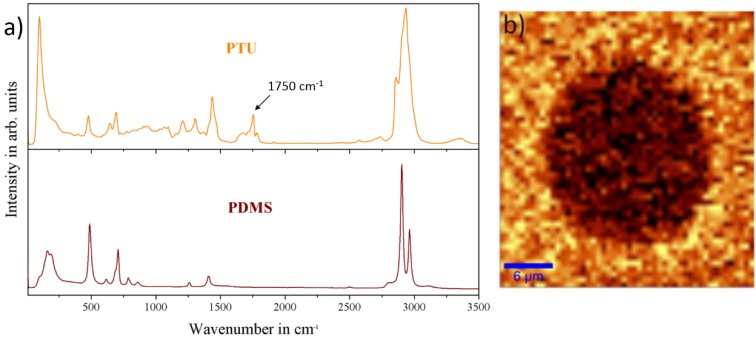
Visualization of separated PTU and PDMS phases by micro-Raman-spectroscopy. (**a**) Micro-Raman spectra of the pure polymers, PTU, and PDMS; (**b**) Filtered micro-Raman image of a microdomain and the surrounding matrix. The PTU signal appears in yellow with respect to the C=O bonding at 1750 cm^−1^, whereas the PDMS signal appears in dark red.

To investigate the chemical composition of the surface in general, and to answer the question of whether there was a proper phase separation between PDMS and PTU, leading to microdomains, and whether there was a chemical interaction between the two polymers, micro-Raman spectra of the composites were taken and compared to the spectra of the respective pure polymers (Figure 2).

The differentiation between the phase-separated polymers was made with respect to the C=O bonding at Raman shift 1750 cm^−1^ [42], which only appears for PTU, and can be thereby clearly distinguished from the signal originating from PDMS. Regarding the area scan, it was shown that the Raman signals of PTU and PDMS were clearly separated from each other in the area where the PDMS formed microdomains within the surrounding PTU matrix (Figure 2b).

This phase separation of the two polymers formed a polymer blend. This can be attributed to the immiscibility of the individual components [21]. One possible explanation for this effect is the difference in surface free energy of PDMS (γ^p^: 0.0 mN/m) and PTU (γ^p^: 12.4 mN/m) (Table 1). This means, for the PDMS component, less favorable thermodynamic conditions for forming interfaces with PTU, as with mostly apolar air [24,25,43,44,45,46,47]. The other factor can be ascribed to the differences in density of PDMS (1.09 g/cm^3^) and PTU (1.2 g/cm^3^), which caused an upwelling force and led to the distribution of PDMS on PTU surface.

### 3.3. Characterization of the Influence of the Addition of t-ZnO Particles on Mechanical Properties of the PTU/PDMS Polymer Blend

**Figure 3:**
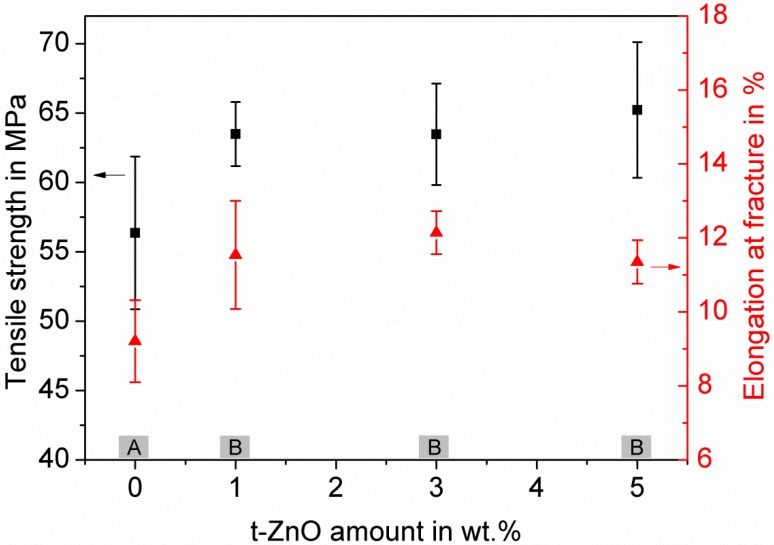
Characterization of tensile strength and elongation at fracture of PTU/PDMS composites with 1 wt.% PDMS and varying content of t-ZnO particles as an additive. Mean values and corresponding standard deviation of tensile strength and elongation at fracture are shown as a function of t-ZnO amount. For statistical analysis of the mean values of tensile strength and elongation at fracture, a one-way ANOVA, followed by a Tukey test, was performed. This showed significant differences between Group A and Group B (marked in the graph), with *p* < 0.05 for both mechanical properties.

The mechanical properties of the PTU/PDMS composites incorporated with different amounts of t-ZnO were evaluated by tensile testing (Figure 3).

The results revealed a remarkable influence of the t-ZnO incorporation on the composites’ mechanical properties, as the mean tensile strength was increased by almost 20%, the elongation at fracture achieved an enhancement of more than 30% for all variations in particle amounts. These data show that the presence of t-ZnO as a filler particle in the composite improves its mechanical properties enormously, but the quantity of particles (1, 3 or 5 wt.%) is only of secondary importance. These assumptions were verified by statistical data analysis (one way ANOVA, Tukey test, *p* < 0.05). For both mechanical properties (tensile strength and elongation at fracture) there was only a significant difference for the filling factors of 0 wt.% t-ZnO and 1 wt.% t-ZnO (Figure 3, group A), higher filler amounts showed no differences in those parameters (Figure 3, group B). Therefore, a t-ZnO particle amount of 1 wt.% within the PTU/PDMS composite was considered as optimum amount to gain outstanding mechanical features and to maintain sufficient processability at best cost-efficiency.

It was already shown by Niu et al. [48] that the incorporation of t-ZnO in resin composites with an amount of up to 5 wt.% causes enhanced tensile properties, whereas further increase of the t-ZnO content, of up to 10 wt.%, weakens mechanical properties. The enhancement effect was ascribed to the special particle geometry which allows for an enhanced uniformity of stress distribution, and therefore yields an increased network stability where the t-ZnO in resins can also prevent the crack propagation, due to their tetrapodal structure with four arms pointing to four different directions. However, increasing the particle content up to 10 wt.% t-ZnO leads to an increase in air bubble inclusions and the higher amount of t-ZnO tends to agglomerate, which then leads to stress concentration and weaker mechanical properties [48]. In our composite, t-ZnO content of 1 wt.% already caused an improvement in the mechanical properties. A further increase in particles up to 5 wt.% did not influence these properties in a strong manner, which seems to contradict other studies [48,49,50]. Previous research [49] has shown that high amount of flower-like ZnO filler above 1 wt.% may agglomerate and give rise to the formation of air bubbles in the composite, which leads to a decrease of the tensile strength. For our results, it can be assumed that one possible effect contributing to this observation might be the influence of the mechanical mixing of the ceramic tetrapodal particles into the polymer. This treatment most likely leads to some damaged tetrapods, exposing highly reactive surfaces. Those surfaces have the potential to form strong adhesion between the particle and the polymer matrix, possibly superimposing the weakening effects due to the higher filler amount.

### 3.4. Effect of t-ZnO Addition on PDMS Microdomain Formation

**Figure 4:**
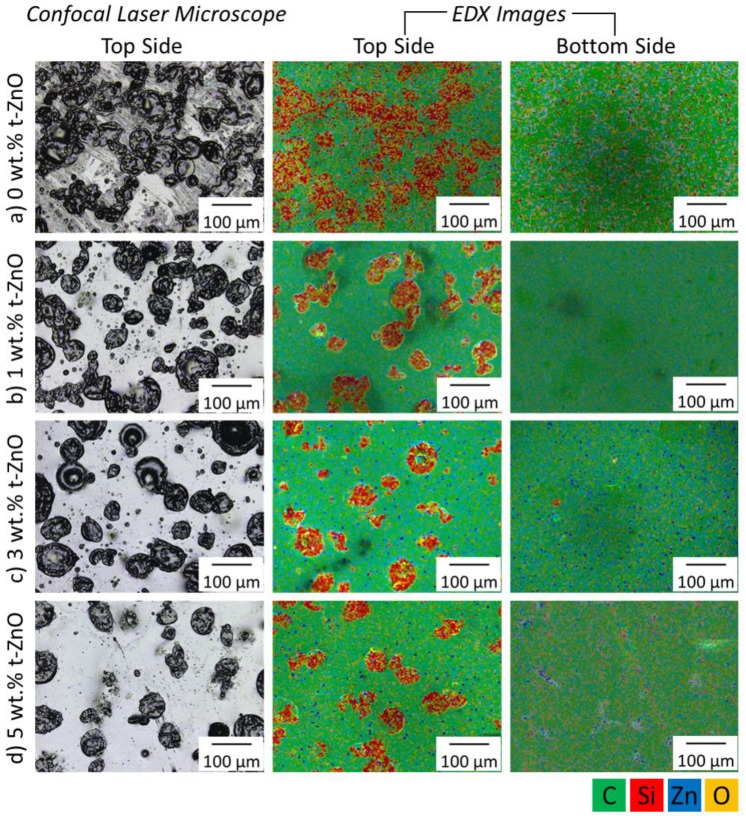
Confocal laser microscopy and energy-dispersive X-ray (EDX) investigation of PTU/PDMS composites with varying t-ZnO particle amounts. (**a**) 0 wt.% t-ZnO, (**b**) 1 wt.% t-ZnO, (**c**) 3 wt.% t-ZnO, (**d**) 5 wt.% t-ZnO. Coding for EDX images: green refers to carbon, red to silicon, blue to zinc, and yellow to oxygen.

The influence of the t-ZnO addition on PDMS domain size and distribution was investigated by confocal laser microscopy and EDX measurements. To evaluate the phase separation and distribution of PDMS and PTU, the top and bottom side of the samples were investigated (Figure 4).

All variations of t-ZnO particle amounts in PTU/PDMS show microdomains of sizes in the range of 50–200 µm. PTU/PDMS composites without additional t-ZnO particles showed inhomogeneous and indistinguishable silicone domains at the surface. The addition of only 1 wt.% t-ZnO had a significant influence on formed domains, as they are more distinct and homogeneously distributed. With 3 wt.% t-ZnO, the homogeneity could again be increased and all microdomains appeared clearly separated at the composites surface. A further increase of the t-ZnO content to 5 wt.% caused less silicone microdomains where only the larger sized domains exist. EDX investigations showed that on the bottom, almost no silicon signal was detected on all samples, which underlines the assumption that the silicone is only present at the sample surfaces. In accordance with increasing t-ZnO content, the zinc signal on the sample bottom increased.

To understand the increased homogeneity of the silicone domains with increased t-ZnO incorporation, the particles can be considered dispersion agents which promote the separation of the individual PDMS domains during the mixing and curing processes. Previously, it was shown that the incorporation of t-ZnO into pure PTU induced the reduction of the polar part of the surface free energy which resulted in decreased wettability of PTU by water [37]. Therefore, one can conclude that the incorporation of t-ZnO into a PTU/PDMS composite leads to the reduction of the driving force for the phase separation of PDMS and PTU-matrix and the differences in materials’ polarity, which consequently favors the more homogeneous distribution of PDMS. The re-agglomeration of the domains at the composite surfaces is impeded by the higher viscosity and the decreased polymerization time because of ceramic particles.

### 3.5. Influence of t-ZnO Addition on Surface Wettability of the PTU/PDMS Polymer Blend

**Figure 5:**
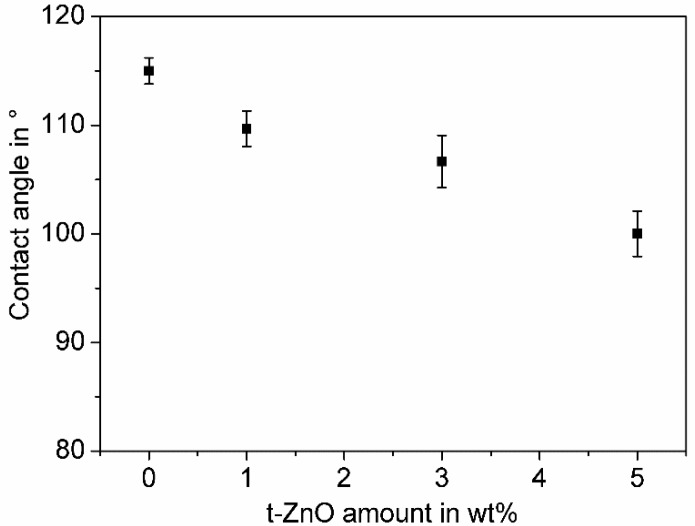
Influence of t-ZnO addition on surface wettability of PTU/PDMS composites with 1 wt.% PDMS. Mean values and corresponding standard deviation of the water contact angle are shown as a function of t-ZnO amount. Mean values and standard deviations are shown.

The effect of t-ZnO addition on the wettability of the polymer composite variations’ surfaces was investigated by water contact angle measurements. The results are shown in the Figure 5.

**Figure 6:**
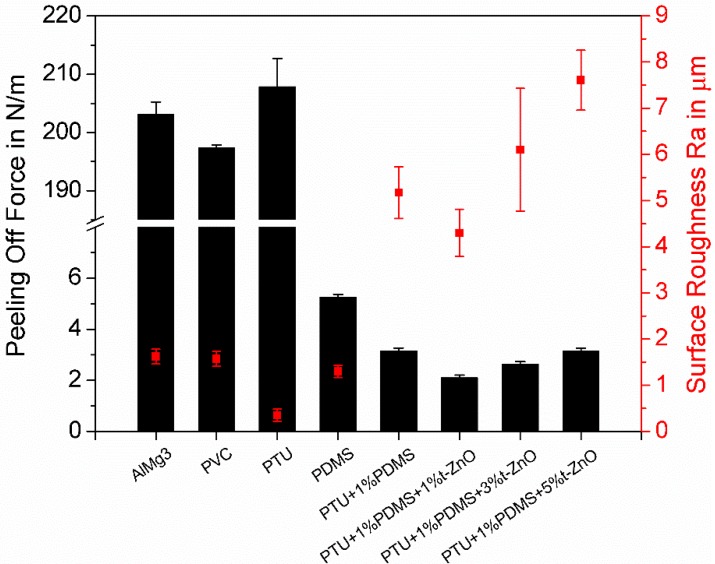
Peel-off force of adhesive tapes from AlMg3, PVC, pure PTU, PDMS, and PTU/PDMS composites with variations of the t-ZnO amount and the corresponding surface roughness.

All composite variations show hydrophobic wetting properties. Without additional t-ZnO, the contact angle measurements of PTU with 1 wt.% PDMS show the highest value, whereby the addition of 1 wt.%, 3 wt.%, and 5 wt.% t-ZnO caused a continuous decrease in the contact angle. At first sight, this finding seems to be contradictory to the observed increase in surface roughness (Figure 6), as in general an increase in surface roughness of hydrophobic material will lead to higher contact angle values [51]. But the increasing amount of t-ZnO in the polymer composite results in distinct and reduced numbers of PDMS domains on the surface (Figure 4) and therefore, as the space in between the hydrophobic domains increases, as does the chances of the water droplet coming into contact with the hydrophilic matrix polymer, and so PTU also increases. In this specific case, the effect of changes in the ratio of the compound’s hydrophobic and hydrophilic surface materials (PDMS and PTU, respectively) do have a bigger impact on the water contact angle than the changes in surface roughness.

### 3.6. Characterization of the Fouling-Release Properties of the Composites by Peel-Off Test

The surface fouling-release properties of PTU/PDMS/t-ZnO composites were investigated under laboratory conditions by peel-off tests compared to pure PTU, PDMS, AlMg3, and PVC as reference surfaces (Figure 6).

The peel-off forces from AlMg3, PVC, and pure PTU are all in the range between 197 N/m and 207 N/m, where the adhesion to pure PTU is higher than those from AlMg3 and PVC. This coincides with the results from the surface roughness Ra measurements, where pure PTU samples show the lowest roughness. As for the pure PDMS surface, which has the similar surface roughness to those of AlMg3 and PVC, the peel force is only 5.3 N/m. This can be explained by the lower surface free energy of PDMS compared to the AlMg3 and PVC surfaces.

Meanwhile, PTU with addition of 1 wt.% PDMS for peel-off force shows the same order of magnitude and even lower values as pure PDMS. The lower peel-off force from PTU/PDMS composites compared to pure PDMS can be explained by the formation of the silicone domains on the composites surfaces, which increases the surface roughness and therefore decreases the contact area. The incorporation of 1 wt.% t-ZnO into PTU/PDMS composites leads to further decrease in peel-off force compared to that without t-ZnO, which can be attributed to the more homogeneously distributed silicone domains and the reduction of the polar part of the surface free energy by an addition of 1 wt.% t-ZnO [37]. The peel-off forces increases slightly with increasing amount of t-ZnO (3 wt.% and 5 wt.%), this might be due to the reduced number of silicone domains on the surface and the thereby increased exposed PTU-matrix surface, which shows in general higher peel-off forces than PDMS.

### 3.7. Fouling Release Behavior in Terms of Barnacles Removing

To gain information about the fouling-release properties of the PTU/PDMS/t-ZnO composites and the formulation PTU/1 wt.% PDMS with 1 wt.% t-ZnO, the reference samples (AlMg3, PVC, pure PTU and pure PDMS) were exposed to the Baltic Sea at Laboe harbor in Laboe, Germany, for a long-term static biofouling field experiment, followed by a barnacle removing study.

**Figure 7:**
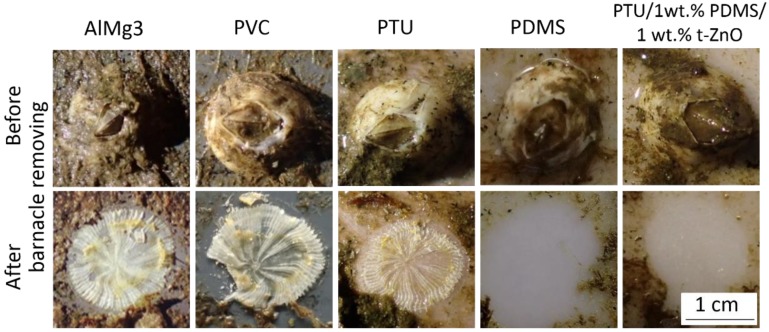
Comparison of test surfaces, before and after barnacle removal. For the PDMS and PTU/1 wt.% PDMS/1 wt.% t-ZnO sample surface, residue free removal without surface damage. In contrast, the AlMg3, PVC, and pure PTU samples showed barnacle residues which could not be removed from the surfaces manually. Scale in all images is identical.

After twelve months of immersion, all sample surfaces showed fouling of algae, mussels, barnacles, and ascidiacea. Because the immersion test was held under static conditions (almost no shear forces acting on the surface), all sample surfaces revealed comparable fouling degree (supplementary material, Figure S1). As the growth of barnacles on immersed substrates to marine environment, e.g., on ship hulls and underwater equipment is most relevant, the removing of barnacles from the samples surfaces was conducted manually to evaluate the fouling-release properties of different materials. The residues on each surface were recorded by camera (Figure 7).

Stronger forces were required to remove barnacles from AlMg3, PVC, and PTU surfaces compared to that from pure PDMS and PTU/PDMS/t-ZnO composite surfaces. For AlMg3, PVC, and pure PTU, residues of the cemented shell, which enable the attachment of the organisms to the substrates, were left behind after removing the barnacles (Figure 7). In contrast, the barnacles on surfaces of the PTU/PDMS/t-ZnO composite and pure PDMS were easily removed by hand. Even the calcareous residues of the barnacles were completely removed, and no surface damage due to biocorrosion was observed.

In general, AlMg3 and PU are well established materials used in ship building and both are known to bear no advantageous antifouling properties for this type of application [31], but do have very advantageous mechanical properties for maritime applications. In contrast to these materials, pure PDMS exhibited fouling-release properties. This fouling-release property of PDMS was in accordance with the literature and can be attributed to low surface energy and low elastic modulus [16,21,52,53,54,55,56]. The fouling-release properties of PTU/PDMS/t-ZnO can be attributed to its comparable surface free energy to pure PDMS.

Overall, the detailed investigations of surface, mechanical, and biofouling properties underlined that PTU/1 wt.% PDMS composites with 1 wt.% t-ZnO particles possess superior properties for applications as biocorosion resistive fouling-release coatings for maritime purposes, which combines the advantageous mechanical and biocorosion inhibiting properties of PTU with the beneficial surface properties of silicone materials and at the same time overcomes the major drawbacks of silicones, such as low adhesion to the substrate and low mechanical stability due to the modification with t-ZnO. Additionally, this fouling-release composite material is well suited for large scale marine applications, like ship hulls and submerged maritime buildings.

## 4. Conclusions

A mechanically durable and easy to handle fouling-release coating based on polythiourethane (PTU), one-component polydimethylsiloxane (PDMS) modified and reinforced by tetrapodal shaped ZnO particles (t-ZnO) has been successfully fabricated and investigated with respect to surface and mechanical properties as well as on its fouling-release performance in terms of barnacle removing. Long-term static immersion experiments under natural conditions and the subsequent barnacles’ removal procedure revealed the promising fouling-release features of the composites. Additionally, no signs of biocorrosion were found. Overall, we successfully demonstrated the development of an ecofriendly marine fouling-release coating with superior mechanical stability.

## Figures and Tables

**Table 1:** Water contact angle, surface free energy (SFE) and the contribution of the polar (γ^p^) and dispersive (γ^d^) fractions of different polymers. PTU/PDMS composite contains 1 wt.% PDMS.

Polymer Variations	Water Contact Angle (°)	SFE (mN/m)	γ^p^ (mN/m)	γ^d^ (mN/m)
PTU [37]	67.4 ± 3.4	40.7 ± 0.5	12.4 ± 1.7	28.4 ± 1.2
PDMS	111.6 ± 1.1	20.9 ± 0.4	0.0 ± 0.0	20.9 ± 0.4
PTU/PDMS	115.0 ± 1.2	20.9 ± 0.8	0.0 ± 0.1	20.9 ± 0.8

## Supplementary Materials

The following are available online at http://www.mdpi.com/1996-1944/11/12/2413/s1, Figure S1: Photographs of different polymer surfaces after twelve months of immersion in the Baltic Sea.
