# Supplementary figures and images for: Development and Characterization of Mechanically Durable Silicone-Polythiourethane Composites Modified with Tetrapodal Shaped ZnO Particles for the Potential Application as Fouling-Release Coating in the Marine Sector
Source: Materials (Basel). 2018 Nov 29;11(12):2413. doi: 10.3390/ma11122413 (PMC6316896; doi:10.3390/ma11122413)

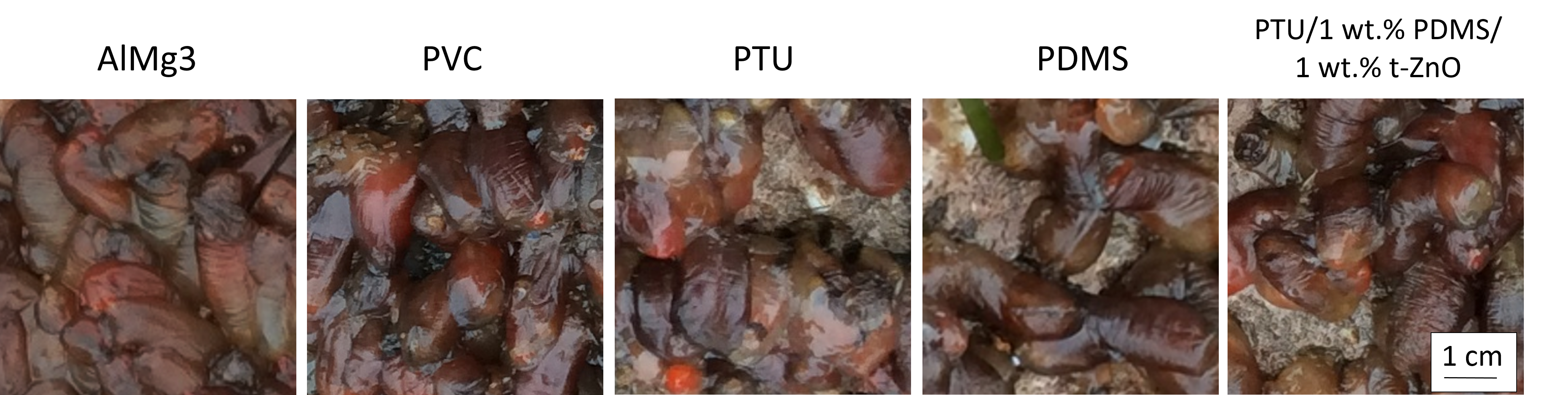

Supplement: Supplementary file 1 [file materials-11-02413-s001.zip › materials-390549-supplementary.tif]
